# Improved Yield of Recombinant Protein via Flagella Regulator Deletion in *Escherichia coli*

**DOI:** 10.3389/fmicb.2021.655072

**Published:** 2021-03-15

**Authors:** Jae-Ho Han, Sang Taek Jung, Min-Kyu Oh

**Affiliations:** ^1^Department of Chemical and Biological Engineering, Korea University, Seoul, South Korea; ^2^BK21 Graduate Program, Department of Biomedical Sciences, College of Medicine, Korea University, Seoul, South Korea

**Keywords:** flagella, recombinant protein, ATP, NADPH, ^13^C metabolic flux analysis

## Abstract

Protein production requires a significant amount of intracellular energy. Eliminating the flagella has been proposed to help *Escherichia coli* improve protein production by reducing energy consumption. In this study, the gene encoding a subunit of FlhC, a master regulator of flagella assembly, was deleted to reduce the expression of flagella-related genes. FlhC knockout in the *ptsG*-deleted strain triggered significant growth retardation with increased ATP levels and a higher NADPH/NADP^+^ ratio. Metabolic flux analysis using a ^13^C-labeled carbon substrate showed increased fluxes toward the pentose phosphate and tricarboxylic acid cycle pathways in the *flhC*- and *ptsG*-deleted strains. Introduction of a high copy number plasmid or overexpression of the recombinant protein in this strain restored growth rate without increasing glucose consumption. These results suggest that the metabolic burden caused by *flhC* deletion was resolved by recombinant protein production. The recombinant enhanced green fluorescent protein yield per glucose consumption increased 1.81-fold in the *flhC* mutant strain. Thus, our study demonstrates that high-yield production of the recombinant protein was achieved with reduced flagella formation.

## Introduction

When a microorganism is used for industrial purposes, its energy metabolism does not have to be similar to that of natural conditions. In an industrial bioreactor, mixing and agitation facilitate nutrient supplements, so that the energy required for movement to search for nutrients would be significantly reduced (Blais et al., 1997). On the other hand, much more energy is needed in the metabolism required for recombinant protein or metabolite production. Although there were attempts to redistribute the energy metabolism optimized for industrial condition through metabolic engineering or genome minimization, it can be said that optimizing complex microorganism metabolism for industrial purposes is still at an early stage (Kolisnychenko et al., 2002; Choe et al., 2019).

The flagellum is a motor that allows bacteria to swim in a liquid and swarm or attach to the surface (Kearns, 2010). *Escherichia coli* typically consists of multiple flagella that make it possible to take action to reach or move away from specific substances (Macnab, 1999). Oxygen and nutrients are the attractants while aromatic or polyamine compounds are the repellents targeted by flagellar movement (Bibikov et al., 1997; Rebbapragada et al., 1997; Bren and Eisenbach, 2000). Flagella contain a thin and long structured filament composed of many proteins (Macnab, 2003). Therefore, a significant amount of energy is needed to synthesize each flagellum and make it move (Macnab, 1996). Under industrial conditions, where agitation or shaking is active, the need for flagella is considerably lower, so the flagella can be removed to save NADPH or ATP. The saved NADPH or ATP can be used in the generation of target products, such as recombinant proteins (Lim et al., 2002; Lee et al., 2007; Kim et al., 2012).

Previously, we constructed glucose transporter mutants, such as *ptsG*, in the *E. coli* W strain and demonstrated that these mutations were beneficial for the improved yield of many target products with reductions in overflow metabolism. When *ptsG* was knocked out, several metabolic flux responses such as reduced glucose uptake rate, upregulated tricarboxylic acid (TCA) cycle, and suppressed acetate production were observed, resulting in an increased yield of the recombinant protein (Jung et al., 2019). In this study, *flhC* was deleted in the constructed *ptsG* knockout mutant. The *flhC* gene encodes a subunit of the FlhC master regulator for flagellar assembly (Fitzgerald et al., 2014; Sim et al., 2017). The *ptsG* and *flhC* double knockout mutants showed growth retardation compared to the *ptsG* knockout mutant. Interestingly, by transfecting a high copy number plasmid into this mutant, the growth rate was restored. The strains were evaluated using ^13^C metabolic flux analysis (^13^C-MFA) in order to explain the restored growth rate and high recombinant protein yield.

## Materials and Methods

### Strains and Plasmids

All strains and plasmids used in this study are listed in Table 1. The host strain in this study was *E. coli* W (KCTC1039), which was provided by the Korean Collection for Type Cultures (KCTC; Jeongeup, Korea). Sugar transporter mutation was performed by deleting *ptsG* (ADT74705), and flagella mutation was performed by deleting *flhC* (ADT75524). All deletion methods utilized λ-red recombinase-based homologous recombination (Datsenko and Wanner, 2000). The name of the *ptsG* knockout mutant was Wp and that of the *flhC* and *ptsG* double mutant was Wpf. All deletions were confirmed via polymerase chain reaction using genomic DNA as a template. All oligonucleotides used in this study were synthesized by Bionics (Seoul, Korea) and are listed in Supplementary Table 1.

**TABLE 1:** Strains and plasmids used in this study.

	Description	References
**Strains**		
W	*Escherichia coli* W KCTC1039	Jung et al., 2019
Wf	W *flhC*:FRT	This study
Wp	W *ptsG*:FRT	This study
Wpf	Wp *flhC*:FRT	This study
WpZ	Wp harboring pZA31 MCS	This study
WpfZ	Wpf harboring pZA31 MCS	This study
WpJ	Wp harboring pJKR-H	This study
WpfJ	Wpf harboring pJKR-H	This study
WpE	Wp harboring pEGFP	This study
WpfE	Wpf harboring pEGFP	This study
**Plasmids**		
pKD46	Red recombinase expression plasmid, *repA*, pSC101ori, PBAD, *gam*, *beta alpha*, *recA*, AmpR	Datsenko and Wanner, 2000
707FLP	Flippase expression plasmid, pSC101ori, *repA*, cl578, FLPe, TetR	Generidge
pZA31 MCS	CmR, p15A ori, PLtetO, rrnB T1	Expressys
pJKR-H	pJKR plasmid with pUC origin	This study
pEGFP	EGFP expression in pZA31 MCS	This study

### Medium and Cultivation

During genetic engineering, strains were cultivated and confirmed in Lysogeny broth (10 g/L of tryptone, 10 g/L of NaCl, and 5 g/L of yeast extract). All cultivations for analysis were performed in M9 minimal medium (6 g/L of Na_2_HPO_4_, 3 g/L of KH_2_PO_4_, 1 g/L of NH_4_Cl, 0.5 g/L of NaCl, and 0.01% Thiamine–HCl) with 20 g/L of glucose and 1 mL of trace elements [2.86 g/L of H_3_BO_3_, 1.81 g/L of MnCl_2_⋅4H_2_O, 0.9 g/L of FeCl_3_⋅6H_2_O, 0.39 g/L of Na_2_MoO_4_⋅2H_2_O, 0.222 g/L of ZnSO_4_⋅7H_2_O, 0.079 g/L of CuSO_4_⋅5H_2_O, and 49.4 mg/L of Co(NO_3_)_2_⋅6H_2_O]. The antibiotics used were carbenicillin (100 μg/mL), kanamycin (50 μg/mL), and chloramphenicol (34 μg/mL). All chemicals were purchased from Sigma-Aldrich (St. Louis, MO, United States).

Cultivation was performed in 250 mL Erlenmeyer flasks with 25 mL of working volume at 250 rpm and 37°C under aerobic conditions.

### Analytical Methods

Bacterial cell mass was estimated via measurement of optical density at 600 nm (OD_600_) using a DU730 (Beckman Coulter, Brea, CA, United States). Measurements of glucose and acetate were performed using high-performance liquid chromatography (Waters, Milford, MA, United States) with a Refractive Index Detector 2414 and SH1011 columns (Shodex, Tokyo, Japan). The measurement was carried out at a temperature of 45°C, and 10 mM sulfuric acid was used as the mobile phase and the flow rate was 0.6 mL/min.

Enhanced green fluorescent protein (EGFP) intensity was measured using a microplate reader (Synergy H1; Biotek, Winooski, VT, United States). The cultured bacterial strains were washed with phosphate-buffered saline (PBS) solution and diluted. The measurement result was multiplied by the dilution ratio. The achieved excitation peak was at 479 nm, and the detected emission peak was at 520 nm.

### Measurements of the ATP/ADP and NADPH/NADP^+^ Ratios

For the ATP assay, BacTiter-Glo^TM^ (Promega, Fitchburg, United States) was used. The culture broth was harvested in the early exponential phase (OD_600__nm_ ≈ 1.0). First, 100 μL of harvested culture broth was mixed with an equal volume of BacTiter-Glo^TM^ reagent. The mixed samples were incubated at room temperature for 5 min. Luminescence was measured using a microplate reader (Synergy H1). The luminescence unit was converted to μM using a standard curve, and the result was divided by dry cell weight. Therefore, the ATP concentration was calculated as μmol/g_cell_.

For the NADPH/NADP^+^ assay, NADP/NADPH-Glo^TM^ (Promega, Fitchburg, United States) was used. Harvested cell of early exponential phase in 50 μL of PBS was lysed in 50 μL of 0.2 M NaOH and 1% (w/v) dodecyl trimethyl ammonium bromide (DTAB). Then, 25 μL of 0.4 M HCl was treated at 60°C for 15 min. Next, 25 μL of 0.5 M Trizma base was added for neutralization, and NADP/NADPH-Glo^TM^ reagents were also added. Samples were incubated at room temperature for 30 min. Luminescence was measured using a microplate reader (Synergy H1). PBS, NaOH, DTAB, HCl, and Trizma base were from Sigma-Aldrich (St. Louis, MO, United States).

### Metabolite Analysis for ^13^C-MFA

For ^13^C-MFA, [1,2-^13^C]-glucose was added to the M9 medium. The 1 mL of cell broth in the early exponential phase (OD_600__nm_ ≈ 1.0) was harvested and centrifuged at 15,000 × *g* for 10 min at 4°C to remove the supernatant. The obtained pellet was washed twice with distilled water and then fully dried with a freeze dryer (Hail, Gimpo, Korea). Then, 200 μL of 6 N HCl was added and hydrolyzed at 110°C for 24 h. After hydrolysis, sample neutralization was performed by adding 200 μL of 6 N NaOH and filtered using an Amicon Ultra 0.5 mL 10 kDa centrifugation filter (Millipore Corporation, Burlington, MA). Next, each sample was derivatized with 80 μL of N-(tert-butyldimethylsilyl)-N-methyl-trifluoroacetamide and 50 μL of pyridine for 1 h at 70°C.

Samples were measured using gas chromatography-mass spectrometry (Agilent 7890 B GC system) with an HP-5MS column (0.25 μm, 30 m × 0.25 mm; Agilent Technology, Santa Clara, CA, United States). The initial temperature was 80°C, which was raised to 280°C at a rate of 7°C/min and held for 10 min. One microliter of the sample was injected at 270°C in 1:10 split mode, and a flow rate of 1 mL/min of helium was used for the mobile phase. The ion source temperature was 230°C, and the electron impact was ionized at 70 eV. Measurement data were analyzed by single ion monitoring, while MassHunter was used for mass spectra, and mass isotopomer distributions (MIDs) were obtained (Antoniewicz et al., 2007a).

### ^13^C-MFA

The network model for the flux analysis construction was conducted based on a previous report (Supplementary Table 2) (Leighty and Antoniewicz, 2013). INCA software, based on the elementary metabolite unit for ^13^C-MFA, was used (Antoniewicz et al., 2007b; Young, 2014). Flux estimation was performed by minimization of the differences between the simulated ones and the MIDs of the proteinogenic amino acids from experimental measurements using least squares regression. Flux estimations were performed 10 times to find the global solution. Chi-square statistical tests were performed for goodness of fit (Im et al., 2020). The metabolic fluxes were calculated from the MIDs of proteinogenic amino acids with an acceptable sum of squared residuals (SSR) (Supplementary Table 3). The SSRs were 13.1 (expected SSR range: 5.6–26.1) and 34.6 (expected SSR range: 16.8–47.0) in Wp and Wpf, respectively.

## Results

### Effects of *flhC* Deletion

Deletion of *flhC* was expected to reduce unnecessary protein production and ATP consumption (Shachrai et al., 2010). First, we deleted the *flhC* gene in the *E. coli* W strain (KCTC1039) and named it Wf. Previously, we demonstrated that the *ptsG* knockout mutant from the W strain (KCTC1039), Wp, showed improved protein production (Jung et al., 2019). Therefore, the *flhC* gene was deleted in the Wp strain to construct the strain named Wpf.

**FIGURE 1:**
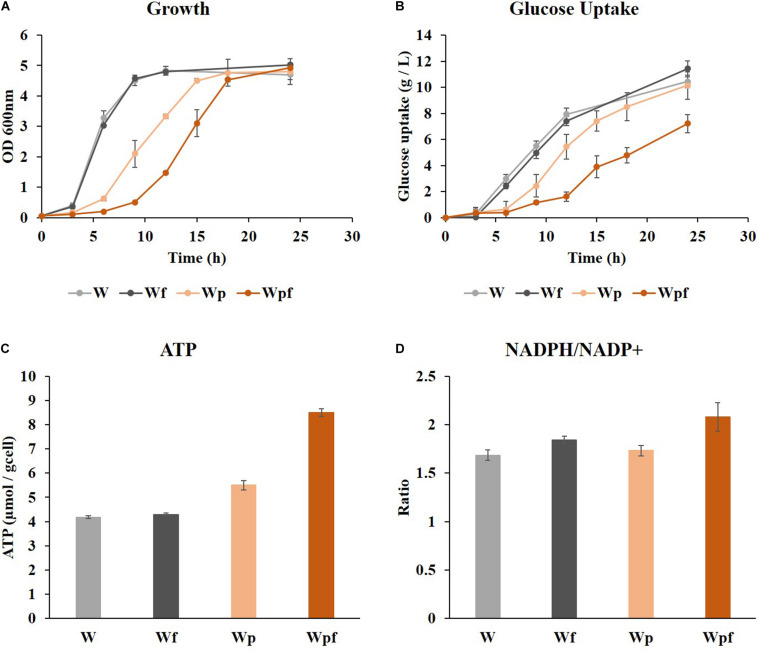
Wild-type (W), *flhC* (Wf), *ptsG* (Wp), and *ptsG/flhC* deletion mutant (Wpf) cultivation in flasks with M9 glucose medium. Growth profiles **(A)**, glucose concentrations **(B)**, specific ATP concentrations **(C)**, and NADPH/NADP^+^ ratios **(D)** of the W, Wf, Wp, and Wpf strains are presented. These results are the average values of the experiment repeated three times.

Cultivation was performed in a shake flask under aerobic conditions in glucose M9 minimal medium at 37°C. Growth retardations with increased lag phase were observed in Wp and Wpf compared to W and Wf (Figure 1A), although the final ODs were similar after 24 h. The amount of glucose consumption was higher in W and Wf, whereas it was significantly decreased in Wpf. In the Wpf strain, the specific glucose uptake rate was also decreased compared to other strains (Figure 1B). These results indicate that Wpf experienced significant metabolic burden by knocking out both *ptsG* and *flhC*. However, the *flhC* knockout strain in the wild-type strain, Wf, did not show any phenotypic differences from the wild-type strain W. To identify the burden, intracellular cofactors in each strain were measured.

ATP levels and NADPH/NADP^+^ ratios were measured to see how these factors were affected by the mutations. Slight increases in the ATP concentration and NADPH/NADP^+^ ratio were observed in the Wf strain compared to those in the W strain (Figures 1C,D). However, the same *flhC* deletion in the *ptsG* knockout mutant caused a much larger effect. The ATP level was increased by 1.5-fold, and the NADPH/NADP^+^ ratio also increased from 1.73 to 2.08 in Wpf compared to Wp. These differences would be significant enough to cause cofactor imbalance, which could result in growth retardation and reduced glucose uptake. The NADH/NAD^+^ ratios were also measured in these strains, which showed little difference between them. To clarify the cofactor imbalance in more detail, ^13^C MFA was applied to the Wp and Wpf strains.

### ^13^C-MFA of the *flhC* and *ptsG* Mutant

**FIGURE 2:**
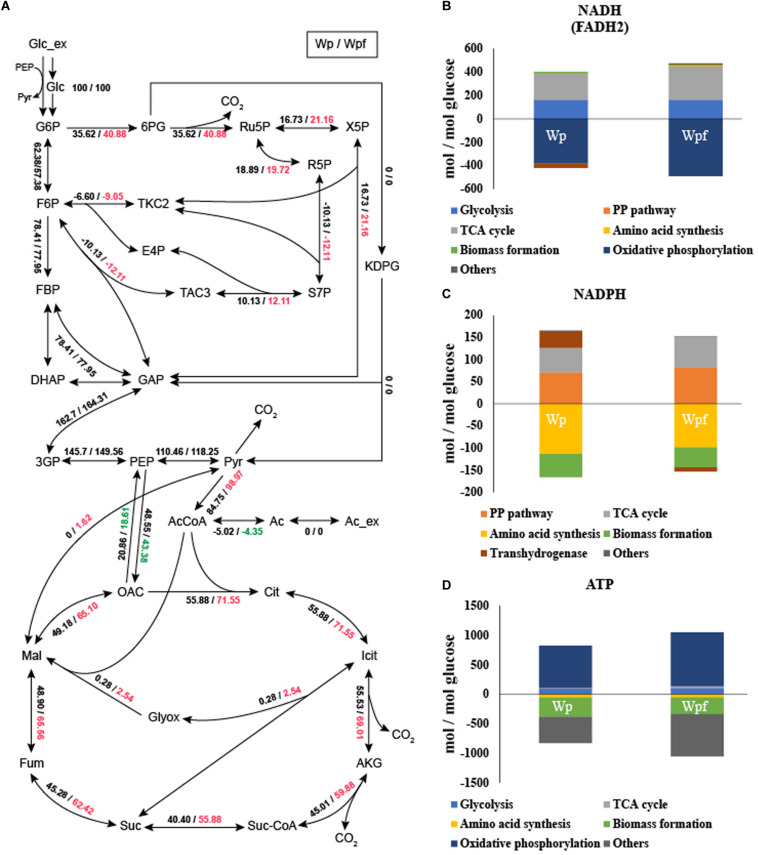
Flux distribution and cofactor balance analysis of the Wp and Wpf strains measured by ^13^C MFA. The relative carbon fluxes of the two strains **(A)** were presented as numbers separated by dashes. The generation (upper) and consumption (lower) pathways of NADH (FADH_2_) **(B)**, NADPH **(C)**, and ATP **(D)** were calculated by balance analysis. These results are the average values of the three different culture broths. Numbers marked in red and green indicate a value that has increased or decreased by more than 10% compared to Wp strain, respectively.

^13^C-MFA was conducted to investigate the metabolic burden of the Wpf strain. The metabolic fluxes of Wpf were compared to those of Wp. The fluxes of the glycolytic pathway were very similar between the two strains. And the fluxes of Entner Doudoroff pathway from 6-phosphogluconate to 2-Keto-3-deoxy-6-phosphogluconate (KDPG) were zero at both two strains. However, the fluxes of the pentose phosphate pathway were changed (Figure 2A). The flux from 6-phosphogluconate to ribose-5-phosphate, the first step in the pentose phosphate pathway, increased from 35.62 to 40.88 in Wpf compared to Wp, and all other fluxes of the subsequent pentose phosphate pathway were increased. Other significant changes in central carbon metabolic fluxes were observed in the TCA pathway. For example, the flux of acetyl CoA to citrate, the first step of the TCA cycle, increased from 55.88 to 71.55 in Wpf compared to Wp, and the fluxes of all other TCA cycle pathways increased in Wpf compared to Wp (Figure 2A).

Based on the carbon flux results, cofactor generation and consumption were also calculated (He et al., 2014; Long et al., 2017, 2018). The results are also showed in Supplementary Table S4. With the increased TCA cycle and pentose phosphate pathway fluxes, the fluxes of NADH (FADH_2_) and NADPH generation also increased 1.28- and 1.2-fold, respectively (Figures 2B,C). Increased NADH levels were used for oxidative phosphorylation. However, despite the increase in NADPH, the NADPH consumption flux for amino acid synthesis and biomass formation decreased by 0.87-fold. Due to this cellular redox imbalance, the flux of transhydrogenase was also changed (Canonaco et al., 2001). In the case of Wp, the flux for converting NADH to NADPH was 38.0173, whereas for Wpf, the flux for converting NADPH to NADH was observed to be 9.1855 (Figures 2B,C). The significant change in transhydrogenase flux was due to cellular redox imbalance, which might be caused by the excessive accumulation of NADPH in the Wpf strain by not forming flagella.

In addition, due to the increased TCA cycle flux, the ATP generation flux of the TCA cycle changed (Figure 2D). It increased 2.27-fold in Wpf compared to Wp. Subsequently, the increased cofactors were converted to ATP by oxidative phosphorylation. The flux of ATP generated by oxidative phosphorylation increased 1.28-fold in Wpf compared to Wp. Finally, at the section of others that are said to be surplus ATP, the flux increased 1.63-fold in Wpf compared to Wp. Large amounts of ATP resulted from deleting *flhC*.

The results of ^13^C-MFA similarly explain the changes in ATP levels and NADPH/NADP^+^ ratios shown in Figures 1C,D. A previous report showed that artificially increased intracellular ATP retarded cell growth (Na et al., 2015). Thus, we suspected that Wpf with high levels of ATP and NADPH induced growth retardation and reduced glucose consumption.

### Growth Restoration by Plasmid Harboring and Overcoming the Metabolic Burden

**FIGURE 3:**
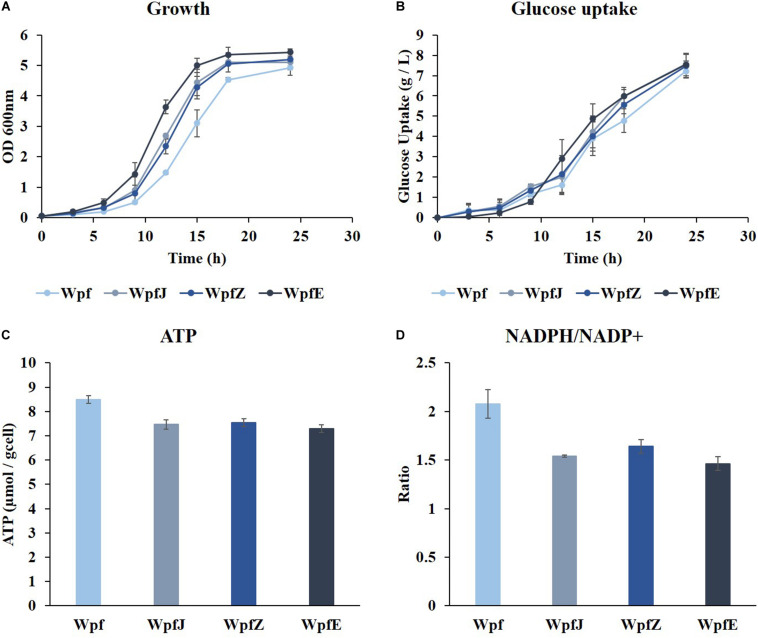
The cultivation results of Wpf, the pJKR-H-harboring strain (WpfJ), the pZA31 MCS-harboring strain (WpfZ), and the pEGFP-harboring strain (WpfE) in flasks with M9 glucose medium. Growth profiles **(A)**, glucose concentrations **(B)**, specific ATP concentrations **(C)**, and NADPH/NADP^+^ ratios **(D)** of the Wpf, WpfJ, WpfZ, and WpfE strains are presented. These results are the average values of the experiment repeated three times.

To reduce the metabolic burden caused by surplus production of ATP and NADPH in the Wpf strain, plasmid replications and protein overexpression were attempted. Plasmids pJKR-H and pZA31 MCS were transformed into the Wpf strain, and the resulting strains were named WpfJ and WpfZ, respectively. In addition, a gene encoding GFP was cloned into pZA31 MCS, and the resulting plasmid was transformed into Wpf, constructing the WpfE strain. The strains were cultivated in a shake flask in M9 glucose medium at 37°C. All three strains, WpfJ, WpfZ, and WpfE, showed growth restoration compared to the Wpf strain (Figure 3A). The growth restoration effect was most significant in the WpfE strain. Interestingly, glucose uptake patterns of those strains were about the same as the Wpf strain, while having much lower glucose uptake than the Wp strain (Figures 1B, 3B).

To investigate the metabolic burden of these strains, ATP levels and NADPH/NADP^+^ ratios were measured. The ATP levels decreased by 0. 88-, 0. 89-, and 0.86-fold in WpfJ, WpjZ, and WpfE, respectively, compared to Wpf (Figure 3C). The NADPH/NADP^+^ ratios also decreased from 2.08 in the Wpf strain to 1.54, 1.64, and 1.46 in WpfJ, WpjZ, and WpfE, respectively (Figure 3D). It is likely that the growth of the Wpf strain was recovered due to the consumption of excess ATP and NADPH by plasmid replication and heterologous protein expression.

### Recombinant Protein Production by the Flagella Knockout Mutant

The excess ATP and NADPH accumulation in cells can be beneficial for recombinant protein production (Lim et al., 2002; Lee et al., 2007; Kim et al., 2012). For example, excess ATP and NADPH were synthesized with a reduced amount of glucose consumption in the Wpf strain. When heterologous protein was expressed in the Wpf strain, EGFP was produced without increased glucose consumption. This means that the yield of protein synthesis per unit of glucose can be increased significantly in the Wpf strain. To verify the effect of *flhC* knockout on the yield of protein synthesis, the WpE and WpfE strains were constructed by transforming the plasmid-expressing EGFP into the Wp and Wpf strains, which were cultivated in a shake flask in glucose M9 medium at 37°C.

**FIGURE 4:**
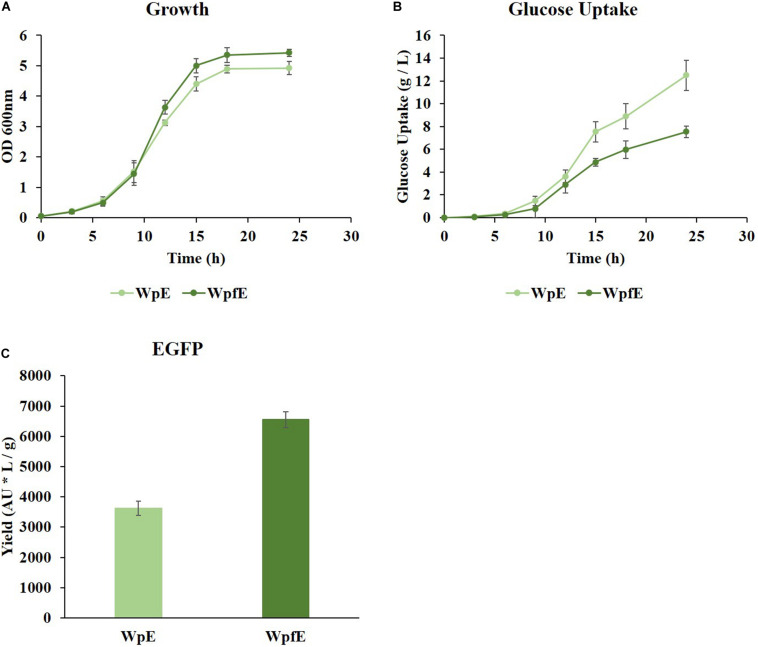
The cultivation results of WpE and WpfE in flasks with M9 glucose medium. The growth profiles **(A)**, glucose concentrations **(B)**, and yields of EGFP production **(C)** are presented. Each yield was calculated by dividing the fluorescence intensity (AU) of EGFP by the glucose consumption (g/L) at 24 h. These results are the average values of the experiment repeated three times.

While growth was restored in WpfE compared to Wpf (Figure 3A and Supplementary Figure 1A), it was decreased in WpE compared to Wp (Supplementary Figure 1B). The growth rate of WpfE was comparable to that of WpE with significantly lower glucose consumption (Figures 4A,B). The glucose uptake decreased from 12.49 g/L to 7.54 g/L in WpfE compared to WpE, despite the similar cell densities reached in both strains. These results suggest that the metabolism of WpfE was much more efficient than that of WpE in order to produce biomass and protein with a unit of glucose. The EGFP yield per consumed glucose increased by 1.81-fold in WpfE compared to WpE (Figure 4C). The WpfE strain showed even higher protein production yield than Wp, which outperformed the wild-type strain in terms of protein yield (Jung et al., 2019).

## Discussion

In this study, flagella were knocked out to construct an efficient *E. coli* strain for energy utilization. First, the *ptsG* knockout mutant Wp, confirmed to have increased production of recombinant protein, was constructed (Jung et al., 2019). Additionally, the *flhC* flagella-related gene was deleted from Wp to create the Wpf strain. Growth retardation was observed in the Wpf strain compared to that in Wp. Using cofactor measurement and ^13^C MFA, the accumulation of ATP and NADPH saved from flagella assembly was found to be the reason. To address this, several plasmids were transformed to consume ATP and NADPH, and growth was restored (Kaleta et al., 2013). Moreover, when a protein was overexpressed using the plasmids, the protein production yield increased. This result contradicts previous studies showing that a metabolic burden arises when a wild-type strain harbors a plasmid (Diaz Ricci and Hernández, 2000; Englaender et al., 2017). Our strategy can be seen as overcoming the growth retardation and inhibition caused by protein expression or high-copy plasmids that introduced a metabolic burden via flagella deletion (Diaz Ricci and Hernández, 2000; Englaender et al., 2017). In particular, the ^13^C MFA results and cofactor measurements suggested the metabolic burden and helped to find a strategy to resolve the problem.

Interestingly, no significant differences in phenotypes were found when *flhC* was knocked out in the wild-type strain. Large amount of ATP level increase and growth retardation and restoration by *flhC* knockout occurred only in the *ptsG* knockout strain. We assumed that the reason for this phenomenon is the C-reactive protein (CRP) mechanism. Previous research has revealed that flagellar biosynthesis is activated by CRP-cAMP (Zhao et al., 2007; Fahrner and Berg, 2015). CRP is activated by binding with cAMP when a preferred carbon source, such as glucose, is depleted (Ishizuka et al., 1994). In the *ptsG*-deleted strain, glucose transport by PTS decreases, and CRP activity rises (Steinsiek and Bettenbrock, 2012). Therefore, in *ptsG* knockout mutants, flagella synthesis and activities may be increased compared to the wild-type strain. In this situation, *flhC* deletion can result in energy saving and significant differences in cell growth and cofactor levels. Because of the beneficial effect of *ptsG* deletion, the mutant is being used more and more widely in industrial strain development (Chou et al., 1994; Chatterjee et al., 2001; Wang et al., 2006). This experiment provides a useful strategy for metabolic engineering based on the *ptsG* mutant.

The *flhC* deletion in the *ptsG* mutant restored the growth rate but not glucose consumption. As a result of ^13^C MFA, the *flhC* deletion in the *ptsG* mutant redistributed the carbon flux from glucose toward the pentose phosphate and TCA cycle pathways. Because more ATP and NAD(P)H could be produced from the same amount of glucose, it was thought that the uptake of glucose was reduced. However, the mechanism of flux redistribution remains unclear. One possibility is that *flhC* is known to regulate the MglBAC transporter (Prüß et al., 2001), which is one of the major glucose uptake transporters in *ptsG* mutants (Nikolic et al., 2013). Therefore, glucose uptake may be reduced by suppressing MglBAC expression through *flhC* deletion.

In conclusion, metabolic perturbation through genetic engineering related to glucose transporter and flagella synthesis was studied, and a strategy for high-yield production of recombinant proteins was proposed. Previously, the production of recombinant proteins in *ptsG*-deleted strains has been suggested to resolve overflow metabolism and improve production yield. In this experiment, intracellular metabolic flux and cofactor generation were further perturbed by *flhC* deletion, which resulted in an even higher yield of protein production. This result could make a significant contribution to strain development for protein production.

## Data Availability Statement

The raw data supporting the conclusions of this article will be made available by the authors, without undue reservation.

## Author Contributions

J-HH, SJ, and M-KO conceived the idea. J-HH performed the experiment. J-HH and M-KO wrote the manuscript. All authors contributed to the article and approved the submitted version.

## Conflict of Interest

The authors declare that the research was conducted in the absence of any commercial or financial relationships that could be construed as a potential conflict of interest.
