# Supplementary figures and images for: Improved Yield of Recombinant Protein via Flagella Regulator Deletion in Escherichia coli
Source: Front Microbiol. 2021 Mar 15;12:655072. doi: 10.3389/fmicb.2021.655072 (PMC8005581; doi:10.3389/fmicb.2021.655072)

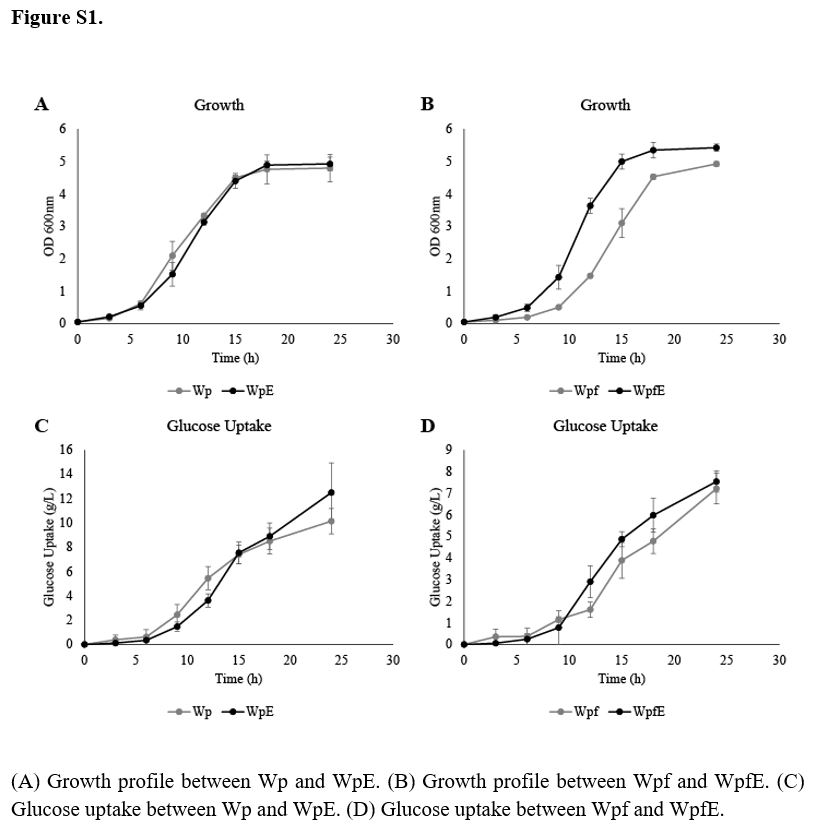

Supplement: Supplementary file 1 [file Image_1.jpg]
