# Supplementary material for: Improved Yield of Recombinant Protein via Flagella Regulator Deletion in Escherichia coli
Source: Front Microbiol. 2021 Mar 15;12:655072. doi: 10.3389/fmicb.2021.655072 (PMC8005581; doi:10.3389/fmicb.2021.655072)
Supplement: Supplementary file 2 [file Table_1.docx]

Improved Yield of Recombinant Protein via Flagella Regulator Deletion in *Escherichia coli*

**Jae-Ho Han ^1^,** **Sang Taek Jung^2^, Min-Kyu Oh ^1^***

^1^ Systems Bioengineering Laboratory, Department of Chemical & Biological Engineering, Korea University, Seoul, Republic of Korea

^2^BK21 Graduate Program, Department of Biomedical Sciences, Korea University College of Medicine, Seoul, 02841, Republic of Korea

^*^Corresponding author:

Min-Kyu Oh

mkoh@korea.ac.kr

Tel./fax.: +82-2-3290-3308

E-mail address: mkoh@korea.ac.kr

Postal address: No. 704 & 705, New Engineering Hall, Dept. of Chemical & Biological Engineering, Korea University, 5-1 Anam-Dong, Sungbuk-Gu, Seoul 02841, Republic of Korea.

**Table S1**. Oligomers used in this study

| Name | Sequence (5` to 3`) | Description |
| --- | --- | --- |
| F_ΔptsG_FKF | AGGTCGGTAAATCGCTGATGCTGCCGGTATCCGTACTGCCTATCGCAGGT gtgtaggctggagctgcttc | FRT-KanR-FRT cassette PCR for *ptsG* mutation |
| R_ΔptsG_FKF | CAGTCGGGTCACCCGCCATATAACGCGGAATGTCGCCGTGGAAAACCTGA gtccatatgaatatcctcct | FRT-KanR-FRT cassette PCR for *ptsG* mutation |
| F_ΔptsG_C | gtaaagttcaccgccgaaaa | Confirmation of *ptsG* mutation |
| R_ΔptsG_C | aatcgcctgaacaccagaac | Confirmation of *ptsG* mutation |
| F_ΔflhC_FKF | TGGGTGGGAATAATGCATACCTCCGAGTTGCTGAAACACATTTATGACATgtgtaggctg gagctgcttc | FRT-KanR-FRT cassette PCR for *flhC* mutation |
| R_ΔflhC_FKF | CAGGCCCTTTTCTTGCGCAGCGCTTCTTCAGGCTGATTAACATCATTCAGgtccatatgaatatcctcct | FRT-KanR-FRT cassette PCR for *flhC* mutation |
| F_ΔflhC_C | ctccgagttgctgaaacaca | Confirmation of *flhC* mutation |
| R_ΔflhC_C | aaccagtcggttgagaatgg | Confirmation of *flhC* mutation |
| EGFP_Fwd | *ATATATGGTACC* atggtgagcaagggcgag | EGFP ORF insertion in pZA31 MCS plasmid |
| EGFP_Rev | *ATATATGTCGAC* cttctctcatccgccaaaac | EGFP ORF insertion in pZA31 MCS plasmid |

Homology arm sequences for gene deletion are shown in capital. The annealing sequences for PCR amplification are shown in lower case. Sequences for restriction enzyme recognition are shown in italic.
