# Supplementary material for: Improved Yield of Recombinant Protein via Flagella Regulator Deletion in Escherichia coli
Source: Front Microbiol. 2021 Mar 15;12:655072. doi: 10.3389/fmicb.2021.655072 (PMC8005581; doi:10.3389/fmicb.2021.655072)
Supplement: Supplementary file 3 [file Table_2.docx]

**Table S2.** Metabolic network model of *E.coli* used for ^13^C-MFA

| **Glycolysis** | | |
| --- | --- | --- |
| Wp/Wpf | Reaction | Carbon atom rearrangement |
| v1 | Glc +ATP → G6P | abcdef → abcdef |
| v2 | G6P ↔ F6P | abcdef ↔ abcdef |
| v3 | F6P + ATP → FBP | abcdef → abcdef |
| v4 | FBP ↔ DHAP + GAP | abcdef ↔ cba + def |
| v5 | DHAP ↔ GAP | abc ↔ abc |
| v6 | GAP ↔ 3PG + ATP + NADH | abc ↔ abc |
| v7 | 3PG ↔ PEP | abc ↔ abc |
| v8 | PEP → Pyr + ATP | abc → abc |
| **Pentose Phosphate Pathway** | | |
| Wp/Wpf | Reaction | Carbon atom rearrangement |
| v9 | G6P → 6PG + NADPH | abcdef → abcdef |
| v10 | 6PG → Ru5P + CO_2_ + NADPH | abcdef → bcdef + a |
| v11 | Ru5P ↔ X5P | abcde ↔ abcde |
| v12 | Ru5P ↔ R5P | abcde ↔ abcde |
| v13 | X5P ↔ TKC2 + GAP | abcde ↔ ab + cde |
| v14 | F6P ↔ TKC2 + E4P | abcdef ↔ ab + cdef |
| v15 | S7P ↔ TKC2 + R5P | abcdefg ↔ ab+ cdefg |
| v16 | F6P ↔ TAC3 + GAP | abcdef ↔ abc + def |
| v17 | S7P ↔ TAC3 + E4P | abcdefg ↔ abc + defg |
| **Entner-Doudoroff Pathway** | | |
| Wp/Wpf | Reaction | Carbon atom rearrangement |
| v18 | 6PG → KDPG | abcdef → abcdef |
| v19 | KDPG → Pyr + GAP | abcdef → abc + def |
| **TCA Cycle** | | |
| Wp/Wpf | Reaction | Carbon atom rearrangement |
| v20 | Pyr → AcCoA + CO_2_ + NADH | abc → bc + a |
| v21 | OAC + AcCoA → Cit | abcd + ef → dcbfea |
| v22 | Cit ↔ ICIT | abcdef ↔ abcdef |
| v23 | ICIT ↔ AKG + CO_2_ + NADPH | abcdef ↔ abcde + f |
| v24 | AKG → SucCoA + CO_2_ + NADH | abcde → bcde + a |
| v25 | SucCoA ↔ Suc + ATP | abcd ↔ abcd |
| v26 | Suc ↔ Fum + FADH2 | abcd ↔ abcd |
| v27 | Fum ↔ Mal | abcd ↔ abcd |
| v28 | Mal ↔ OAC + NADH | abcd ↔ abcd |
| **Glyoxylate Shunt** | | |
| Wp/Wpf | Reaction | Carbon atom rearrangement |
| v29 | ICIT → Glyox + Suc | abcdef → ab + edcf |
| v30 | Glyox + AcCoA → Mal | ab + cd → abdc |
| **Amphibolic Reactions** | | |
| Wp/Wpf | Reaction | Carbon atom rearrangement |
| v31 | Mal → Pyr + CO_2_ + NADPH | abcd → abc + d |
| v32 | Mal → Pyr + CO_2_ + NADH | abcd → abc + d |
| v33 | PEP + CO_2_ → OAC | abc + d → abcd |
| v34 | OAC + ATP → PEP + CO_2_ | abcd → abc + d |
| **Acetic Acid Formation** | | |
| Wp/Wpf | Reaction | Carbon atom rearrangement |
| v35 | AcCoA ↔ Ac + ATP | ab ↔ ab |
| **Amino Acid Biosynthesis** | | |
| Wp/Wpf | Reaction | Carbon atom rearrangement |
| v36 | AKG + NADPH + NH_3_ → Glu | abcde → abcde |
| v37 | Glu + ATP + NH_3_ → Gln | abcde → abcde |
| v38 | Glu + ATP + 2 NADPH → Pro | abcde → abcde |
| v39 | Glu + CO_2_ + Gln + Asp + AcCoA + 5 ATP + NADPH → Arg + AKG + Fum + Ac | abcde + f + ghijk + lmno + pq → abcdef + ghijk + lmno + pq |
| v40 | OAC + Glu → Asp + AKG | abcd + efghi → abcd + efghi |
| v41 | Asp + 2 ATP + NH_3_ → Asn | abcd → abcd |
| v42 | Pyr + Glu → Ala + AKG | abc + defgh → abc + defgh |
| v43 | 3PG + Glu → Ser + AKG + NADH | abc + defgh → abc + defgh |
| v44 | Ser ↔ Gly + MEETHF | abc ↔ ab + c |
| v45 | Gly ↔ CO_2_ + MEETHF + NADH + NH3 | ab ↔ a + b |
| v46 | Thr → Gly + AcCoA + NADH | abcd → ab + cd |
| v47 | Ser + AcCoA + 3 ATP + 4 NADPH + SO_4_ → Cys + Ac | abc + de → abc + de |
| v48 | Asp + Pyr + Glu + SucCoA + ATP +2 NADPH → LL-DAP + AKG + Suc | abcd + efg + hijkl + mnop → abcdgfe + hijkl + mnop |
| v49 | LL-DAP → Lys + CO_2_ | abcdefg → abcdef + g |
| v50 | Asp + 2 ATP + 2 NADPH → Thr | abcd → abcd |
| v51 | Asp + METHF + Cys + SucCoA + ATP + 2 NADPH → Met + Pyr + Suc + NH_3_ | abcd + e + fgh + ijkl → abcde + fgh + ijkl |
| v52 | Pyr + Pyr + Glu + NADPH → Val + CO2 + AKG | abc + def + ghijk → abcef + d + ghijk |
| v53 | AcCoA + Pyr + Pyr + Glu + NADPH → Leu + CO_2_ + CO_2_ + AKG + NADH | ab + cde + fgh + ijklm → abdghe + c + f + ijklm |
| v54 | Thr + Pyr + Glu + ATP + NADPH → Ile + CO2 + AKG | abcd + efg + hijkl → abfcdg + e + hijkl |
| v55 | PEP + PEP + E4P + Glu + ATP + NADPH → Phe + CO_2_ + AKG | abc + def + ghij + klmno → abcefghij + d + klmno |
| v56 | PEP + PEP + E4P + Glu + ATP + NADPH → Tyr + CO_2_ + AKG + NADH | abc + def + ghij + klmno → abcefhgij + d + klmno |
| v57 | Ser + R5P + PEP + E4P + PEP + Gln + 3 ATP + NADPH → Trp + CO_2_ + GAP + Pyr + Glu | abc + defgh + ijk + lmno + pqr + stuvw → abcedklmnoj + i + fgh + pqr + stuvw |
| v58 | R5P + FTHF + Gln + Asp + 5 ATP → His + AKG + Fum + 2 NADH | abcde + f + ghijk + lmno → edcbaf + hgijk + lmno |
| **One-Carbon Metabolism** | | |
| Wp/Wpf | Reaction | Carbon atom rearrangement |
| v59 | MEETHF + NADH → METHF | a → a |
| v60 | MEETHF → FTHF + NADPH | a → a |
| **Oxidative Phosphorylation** | | |
| Wp/Wpf | Reaction | Carbon atom rearrangement |
| v61 | NADH + 1/2 O_2_ → 2 ATP |  |
| v62 | FADH2 + 1/2 O_2_ → ATP |  |
| **Transhydrogenation** | | |
| Wp/Wpf | Reaction | Carbon atom rearrangement |
| v63 | NADH ↔ NADPH |  |
| **ATP Hydrolysis** | | |
| Wp/Wpf | Reaction | Carbon atom rearrangement |
| v64 | ATP → ATP_EX |  |
| **Transport** | | |
| Wp/Wpf | Reaction | Carbon atom rearrangement |
| v65 | Ac → Ac_EX |  |
| v66 | CO_2_ → CO_2__EX | a → a |
| v67 | O_2__EX → O_2_ |  |
| v68 | NH_3__EX → NH_3_ |  |
| v69 | SO_4__EX → SO_4_ |  |
| **Biomass Formation** | | |
| Wp/Wpf | Reaction | Carbon atom rearrangement |
| v70 | 0.488 Ala + 0.281 Arg + 0.229 Asn + 0.229 Asp + 0.087 Cys + 0.25 Glu |  |
| **CO_2_ exchange** | | |
| Wp/Wpf | Reaction | Carbon atom rearrangement |
| v71 | CO_2__unlabeled + CO_2_ → CO_2_ + CO_2__out | a + b → a + b |
