# Supplementary material for: Improved Yield of Recombinant Protein via Flagella Regulator Deletion in Escherichia coli
Source: Front Microbiol. 2021 Mar 15;12:655072. doi: 10.3389/fmicb.2021.655072 (PMC8005581; doi:10.3389/fmicb.2021.655072)
Supplement: Supplementary file 4 [file Table_3.docx]

**Table S3.** Mass Isotopomer Distributions(MID) from culture broth with [12-^13^C] glucose

| Strain | Wp | Wpf |
| --- | --- | --- |
| Gly246 |  |  |
| m0 | 0.443579 | 0.458363 |
| m1 | 0.38277 | 0.371271 |
| m2 | 0.127096 | 0.12622 |
| m3 | 0.039046 | 0.036934 |
| m4 | 0.007509 | 0.007212 |
| Ala232 |  |  |
| m0 | 0.409233 | 0.418 |
| m1 | 0.169533 | 0.175359 |
| m2 | 0.329902 | 0.31843 |
| m3 | 0.065927 | 0.063941 |
| m4 | 0.025405 | 0.02427 |
| Ala260 |  |  |
| m0 | 0.400421 | 0.407942 |
| m1 | 0.140956 | 0.147848 |
| m2 | 0.334699 | 0.323426 |
| m3 | 0.087925 | 0.085763 |
| m4 | 0.03064 | 0.029697 |
| m5 | 0.005359 | 0.005325 |
| Leu274 |  |  |
| m0 | 0.146921 | 0.146697 |
| m1 | 0.121339 | 0.180316 |
| m2 | 0.260362 | 0.248285 |
| m3 | 0.228806 | 0.211644 |
| m4 | 0.14053 | 0.126991 |
| m5 | 0.080059 | 0.069923 |
| m6 | 0.018522 | 0.016143 |
| m7 | 0.003461 | 0 |
| Ile274 |  |  |
| m0 | 0.16467 | 0.167334 |
| m1 | 0.159736 | 0.170376 |
| m2 | 0.276608 | 0.274005 |
| m3 | 0.187021 | 0.187605 |
| m4 | 0.136044 | 0.129966 |
| m5 | 0.060951 | 0.056668 |
| m6 | 0.01497 | 0.014046 |
| m7 | 0 | 0 |
| Ser362 |  |  |
| m0 | 0.355056 | 0.351138 |
| m1 | 0.236535 | 0.259064 |
| m2 | 0.285845 | 0.272638 |
| m3 | 0.087673 | 0.084909 |
| m4 | 0.034891 | 0.032251 |
| Ser390 |  |  |
| m0 | 0.331387 | 0.327315 |
| m1 | 0.215963 | 0.238136 |
| m2 | 0.298845 | 0.286334 |
| m3 | 0.110282 | 0.107249 |
| m4 | 0.043523 | 0.040966 |
| m5 | 0.010361 | 0.009661 |
| Phe302 |  |  |
| m0 | 0.428286 | 0.437884 |
| m1 | 0.384296 | 0.377537 |
| m2 | 0.138114 | 0.136373 |
| m3 | 0.040812 | 0.040164 |
| m4 | 0.008493 | 0.008043 |
| Phe308 |  |  |
| m0 | 0.154417 | 0.153375 |
| m1 | 0.113858 | 0.118559 |
| m2 | 0.259641 | 0.253528 |
| m3 | 0.158907 | 0.162667 |
| m4 | 0.165615 | 0.16321 |
| m5 | 0.082639 | 0.083832 |
| m6 | 0.042117 | 0.043031 |
| m7 | 0.017946 | 0.017368 |
| m8 | 0.004858 | 0.00443 |
| Asp302 |  |  |
| m0 |  | 0.382502 |
| m1 |  | 0.307862 |
| m2 |  | 0.227453 |
| m3 |  | 0.061278 |
| m4 |  | 0.020905 |
| Asp390 |  |  |
| m0 |  | 0.258738 |
| m1 |  | 0.244699 |
| m2 |  | 0.272848 |
| m3 |  | 0.152524 |
| m4 |  | 0.054396 |
| m5 |  | 0.016796 |
| Asp418 |  |  |
| m0 | 0.230545 | 0.229809 |
| m1 | 0.196193 | 0.20522 |
| m2 | 0.281055 | 0.27514 |
| m3 | 0.162174 | 0.162397 |
| m4 | 0.091766 | 0.089921 |
| m5 | 0.029188 | 0.028616 |
| m6 | 0.009079 | 0.008897 |
| Glu330 |  |  |
| m0 | 0.186031 | 0.190723 |
| m1 | 0.194664 | 0.206002 |
| m2 | 0.289909 | 0.285178 |
| m3 | 0.171762 | 0.171265 |
| m4 | 0.118702 | 0.110488 |
| m5 | 0.029703 | 0.028211 |
| m6 | 0.009229 | 0.008133 |
| Glu432 |  |  |
| m0 | 0.142262 | 0.145937 |
| m1 | 0.154818 | 0.163791 |
| m2 | 0.260663 | 0.259196 |
| m3 | 0.196544 | 0.19634 |
| m4 | 0.146633 | 0.140489 |
| m5 | 0.069111 | 0.066201 |
| m6 | 0.023085 | 0.021737 |
| m7 | 0.006884 | 0.006308 |
| Tyr302 |  |  |
| m0 | 0.428468 | 0.43785 |
| m1 | 0.383745 | 0.37662 |
| m2 | 0.137732 | 0.136319 |
| m3 | 0.040799 | 0.040009 |
| m4 | 0.009257 | 0.009202 |
