# Supplementary material for: Improved Yield of Recombinant Protein via Flagella Regulator Deletion in Escherichia coli
Source: Front Microbiol. 2021 Mar 15;12:655072. doi: 10.3389/fmicb.2021.655072 (PMC8005581; doi:10.3389/fmicb.2021.655072)
Supplement: Supplementary file 5 [file Table_4.docx]

**Table S4.** Results of ^13^C-metabolic flux analysis

| No. | Best fit | |
| --- | --- | --- |
|  | Wp | Wpf |
| R1 | 100 | 100 |
| R2 net | 62.3789 | 57.3847 |
| R2 exch | 176.7961 | 5153400 |
| R3 | 78.4145 | 77.9461 |
| R4 net | 78.4145 | 77.9461 |
| R4 exch | 3.8522 | 1E-07 |
| R5 net | 78.4145 | 77.9461 |
| R5 exch | 0.000124 | 52.0583 |
| R6 net | 162.6995 | 164.3095 |
| R6 exch | 1.23E-06 | 1960900 |
| R7 net | 145.7009 | 149.559 |
| R7 exch | 140510 | 5048900 |
| R8 | 110.4588 | 118.2458 |
| R9 | 35.6202 | 40.8789 |
| R10 | 35.6202 | 40.8789 |
| R11 net | 16.7287 | 21.1627 |
| R11 exch | 73.9444 | 52.7981 |
| R12 net | 18.8915 | 19.7162 |
| R12 exch | 40.9777 | 1785300 |
| R13 net | 16.7287 | 21.1627 |
| R13 exch | 29.581 | 41.989 |
| R14 net | -6.6025 | -9.0525 |
| R14 exch | 5.9867 | 7.8991 |
| R15 net | -10.1262 | -12.1102 |
| R15 exch | 30.194 | 31.106 |
| R16 net | -10.1262 | -12.1102 |
| R16 exch | 125.452 | 96.2862 |
| R17 net | 10.1262 | 12.1102 |
| R17 exch | 15.0324 | 17.7704 |
| R18 | 8.62E-07 | 9.45E-08 |
| R19 | 8.62E-07 | 9.45E-08 |
| R20 | 84.7536 | 98.9724 |
| R21 | 55.8032 | 71.5496 |
| R22 net | 55.8032 | 71.5496 |
| R22 exch | 0.000102 | -1.3E-06 |
| R23 net | 55.5304 | 69.0127 |
| R23 exch | 31896 | 3535600 |
| R24 | 45.0082 | 59.8821 |
| R25 net | 40.3962 | 55.88 |
| R25 exch | 5.5326 | 28606 |
| R26 | 45.281 | 62.419 |
| R27 net | 48.9023 | 65.5613 |
| R27 exch | 0.000106 | 0.000181 |
| R28 net | 49.1752 | 65.066 |
| R28 exch | 27.318 | 1E-07 |
| R29 | 0.2729 | 2.5369 |
| R30 | 0.2729 | 2.5369 |
| R31 | 1.16E-06 | 1.4089 |
| R32 | 1.16E-06 | 1.6233 |
| R33 | 48.5548 | 43.3795 |
| R34 | 20.8578 | 18.6135 |
| R35 net | -5.0171 | -4.3524 |
| R35 exch | 1.0878 | 0.4627 |
| R36 | 63.7434 | 55.313 |
| R37 | 6.5886 | 5.7172 |
| R38 | 2.0498 | 1.7787 |
| R39 | 2.7428 | 2.3801 |
| R40 | 17.7502 | 15.4026 |
| R41 | 2.2352 | 1.9396 |
| R42 | 4.7633 | 4.1333 |
| R43 | 10.9566 | 9.5075 |
| R44 net | 6.1542 | 5.3403 |
| R44 exch | 3.3721 | 5.0804 |
| R45 net | 0.4734 | 0.4108 |
| R45 exch | 0.851 | 589160 |
| R46 | 8.72E-07 | 9.82E-08 |
| R47 | 2.2743 | 1.9735 |
| R48 | 3.1869 | 2.7654 |
| R49 | 3.1869 | 2.7654 |
| R50 | 5.0464 | 4.379 |
| R51 | 1.4251 | 1.2366 |
| R52 | 3.9239 | 3.4049 |
| R53 | 4.1777 | 3.6251 |
| R54 | 2.694 | 2.3377 |
| R55 | 1.7179 | 1.4907 |
| R56 | 1.2787 | 1.1096 |
| R57 | 0.5271 | 0.4574 |
| R58 | 0.8785 | 0.7623 |
| R59 | 1.4251 | 1.2366 |
| R60 | 0.8785 | 0.7623 |
| R61 | 335.0394 | 426.3035 |
| R62 | 45.281 | 62.419 |
| R63 net | 38.0173 | -9.1855 |
| R63 exch | 38.4121 | 0.0767 |
| R64 | 440.0608 | 718.8459 |
| R65 | 1E-07 | 0.0012 |
| R66 | 212.6297 | 263.8591 |
| R67 | 190.1602 | 244.3612 |
| R68 | 70.6688 | 61.3225 |
| R69 | 2.2743 | 1.9735 |
| R70 | 9.7609 | 8.47 |
| R71 | 132.7564 | 450.8205 |
| The proportion of labeled amino acid (G-value) | | |
| Ala | 100 | 99 |
| Gly | 95 | 84 |
| Val | 50 | 50 |
| Leu | 50 | 97 |
| Ile | 98 | 97 |
| Ser | 93 | 92 |
| Phe | 96 | 96 |
| Asp | 96 | 97 |
| Glu | 98 | 96 |
| Tyr | 96 | 95 |
